# The Streets

**Published:** 1902-02

**Authors:** 


### The Streets.

THE streets of Buffalo, for some years past have been the pride of our citizens. To the summer visitors they present an attractive, cleanly appearance, and our 250 miles of smooth pavement make us the envy of all observers from less fortunately paved cities. For about nine months of the year we are entitled, with great justice, to this praise and envy; but what shall we say for the remainder of the year?

In the winter season, with heavy falls of snow at repeated intervals which is scraped into ridges and heaps by the remorselessness of the Street Railway Company, there is little comfort in driving into the business part of the city with any kind of a vehicle, on runners or wheels. Indeed, it is almost impossible to approach a shop on either side of our principal business streets without danger to horses, since they are liable to fall into the gutter.

The mildness of the present winter has developed into frequent thaws, and so this season our bad streets have put in an appearance earlier than usual. The present commissioner of public works offers as an excuse for not cleaning the streets that funds are low early in January, there being but $4,000 left to last out the season. If Colonel Ward is correctly reported he replied, in substance, to a recent question as to why the streets were not cleaned, that it would be foolish to clean Main Street or any other street at this time; that it would be a useless expenditure of money; that a snow storm is likely to come upon Buffalo at any moment and that all the work would have to be done over again, with probably the same result.

Here is logic dealt out in chunks! Don't clean the streets now, because by and by they will get dirty again, so what's the use? We have been treated year by year with the cry "short of funds" by the public works office, but here is a new reason offered by the new commissioner.

We have over and again pointed out in these columns, that the most economical way to deal with the poor is to employ them. We need not repeat the argument here. It ought to be patent to every public official. The streets should be cleaned, likewise on economic grounds. When they are filthy health is endangered; moreover, the thick layers of ice and slush, which alternate as the weather changes, serves to decay the asphalt pavement. Therefore for these two reasons,—economy in health and economy in money,—let us have done with this perennial excuse of no money, and for once have a sufficient sum appropriated to last the season through and give us clean streets. Our contention is that the business streets should be cleaned from curb to curb during every thaw, no matter whether in mid-winter or late spring.

